# Supplementary figures and images for: Evaluation of M2-like macrophage enrichment after diffuse traumatic brain injury through transient interleukin-4 expression from engineered mesenchymal stromal cells
Source: J Neuroinflammation. 2020 Jun 20;17:197. doi: 10.1186/s12974-020-01860-y (PMC7306141; doi:10.1186/s12974-020-01860-y)

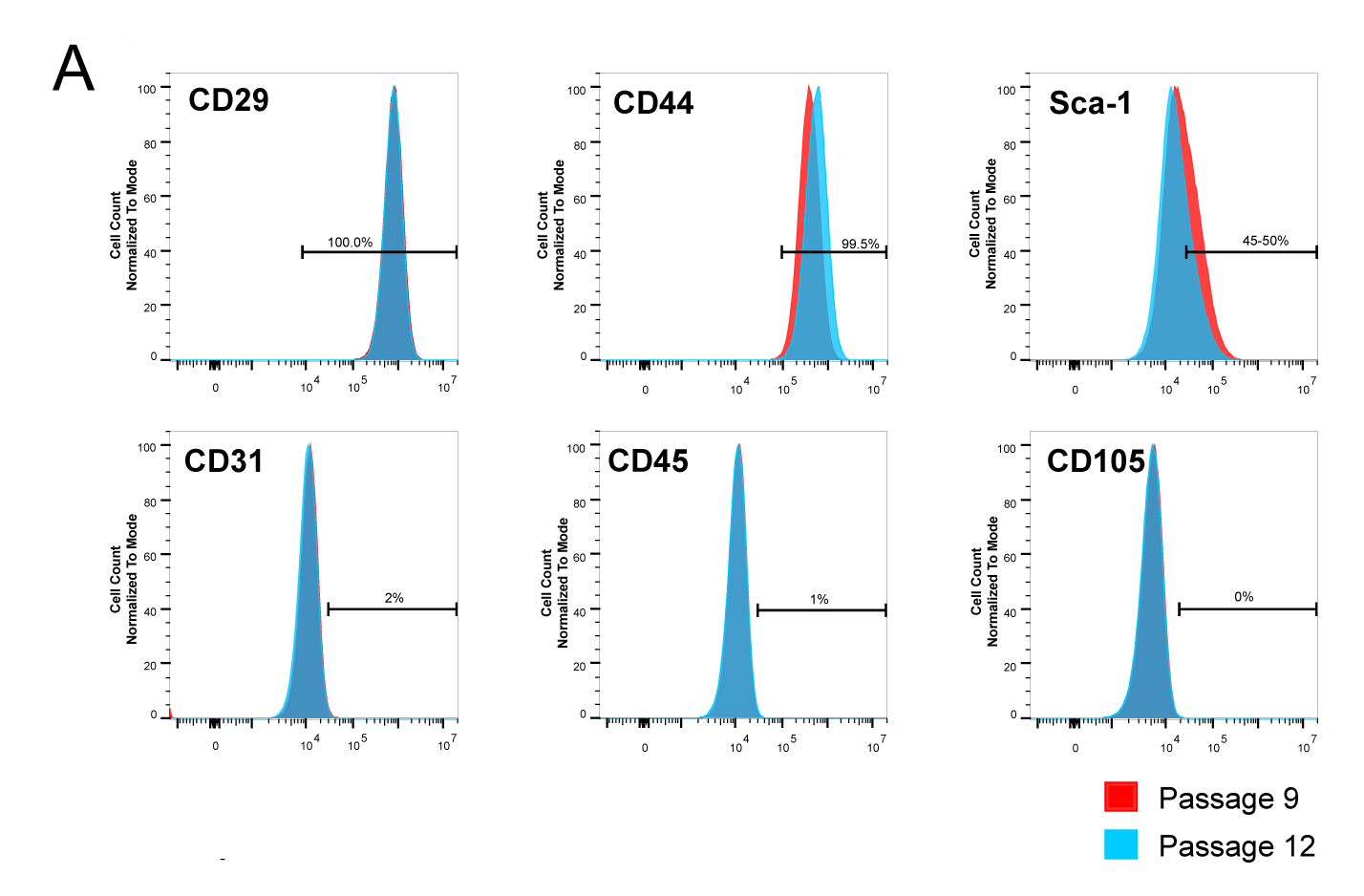

Supplement: Supplementary file 1 — Additional file 1: Figure S1. MSC cell-surface markers. (A) MSCs (Cyagen) used in this study expressed CD29, CD44, and Sca-1 while remaining negative for CD31 and CD45 in at least two late passage numbers. [file 12974_2020_1860_MOESM1_ESM.tif]

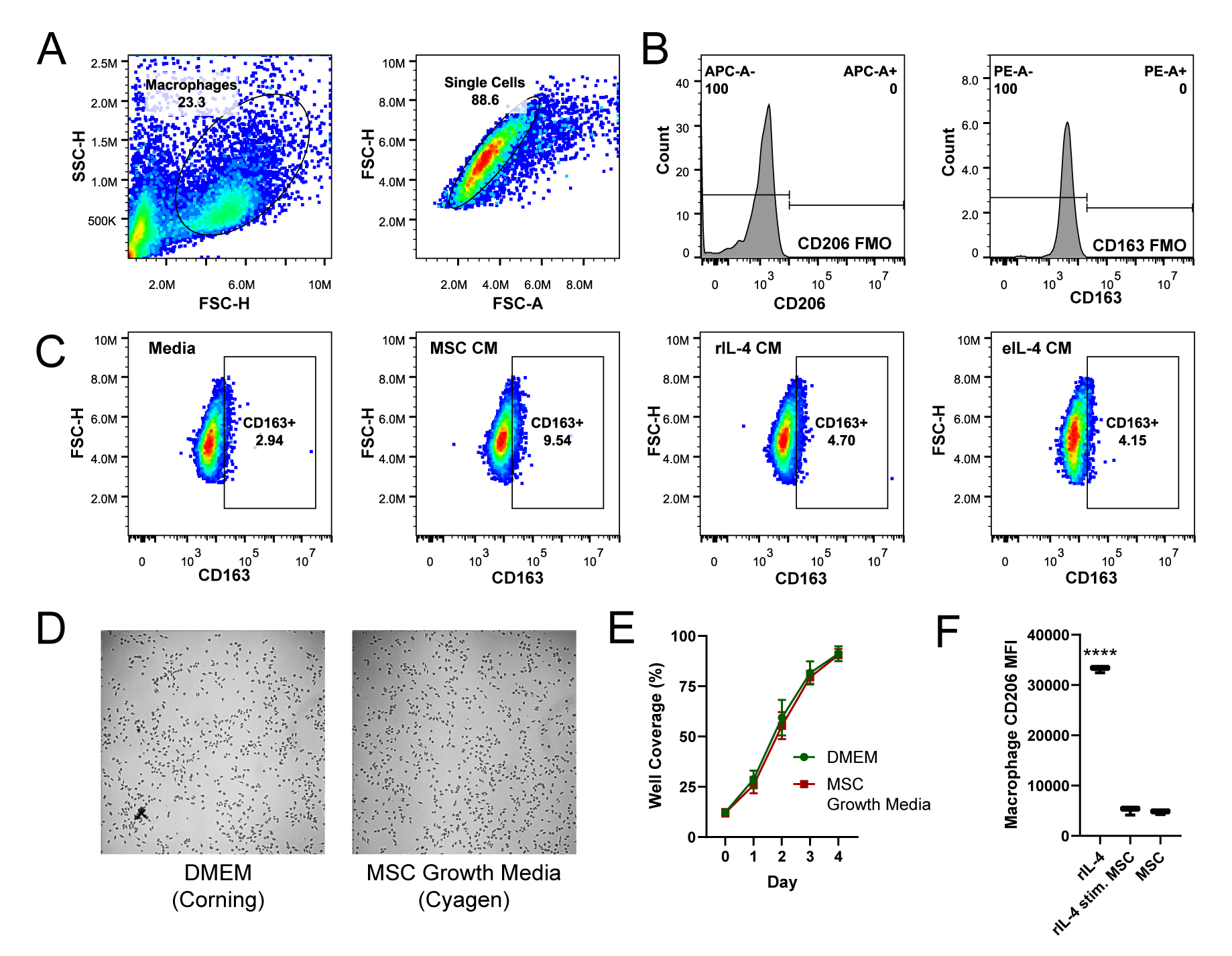

Supplement: Supplementary file 2 — Additional file 2: Figure S2. Macrophage and MSC control experiments. (A) Flow gating strategy of J774A.1 macrophages prior to individual antibody gates. (B) Fluorescence-minus-one plots to determine antibody gates. (C) Flow plots demonstrating CD163 expression after treatment with differently conditioned media. (D) Brightfield images of macrophages grown in their recommended media (Dulbecco’s Modified Eagle Media, DMEM) or MSC growth media. Each image is 1/16th of total image analyzed per well. (E) Growth curves of macrophages in each of the media estimated by coverage of the well, calculated from automated imaging scripts. (F) Mean fluorescence intensity of CD206 expression on macrophages after treatment with either 100 ng/mL recombinant IL-4 (rIL-4), conditioned media from MSCs pre-stimulated with 100 ng/mL rIL-4, or conditioned media from naïve MSCs. Mean ± SD, one- or two-way ANOVA; ****p < 0.0001. [file 12974_2020_1860_MOESM2_ESM.tif]

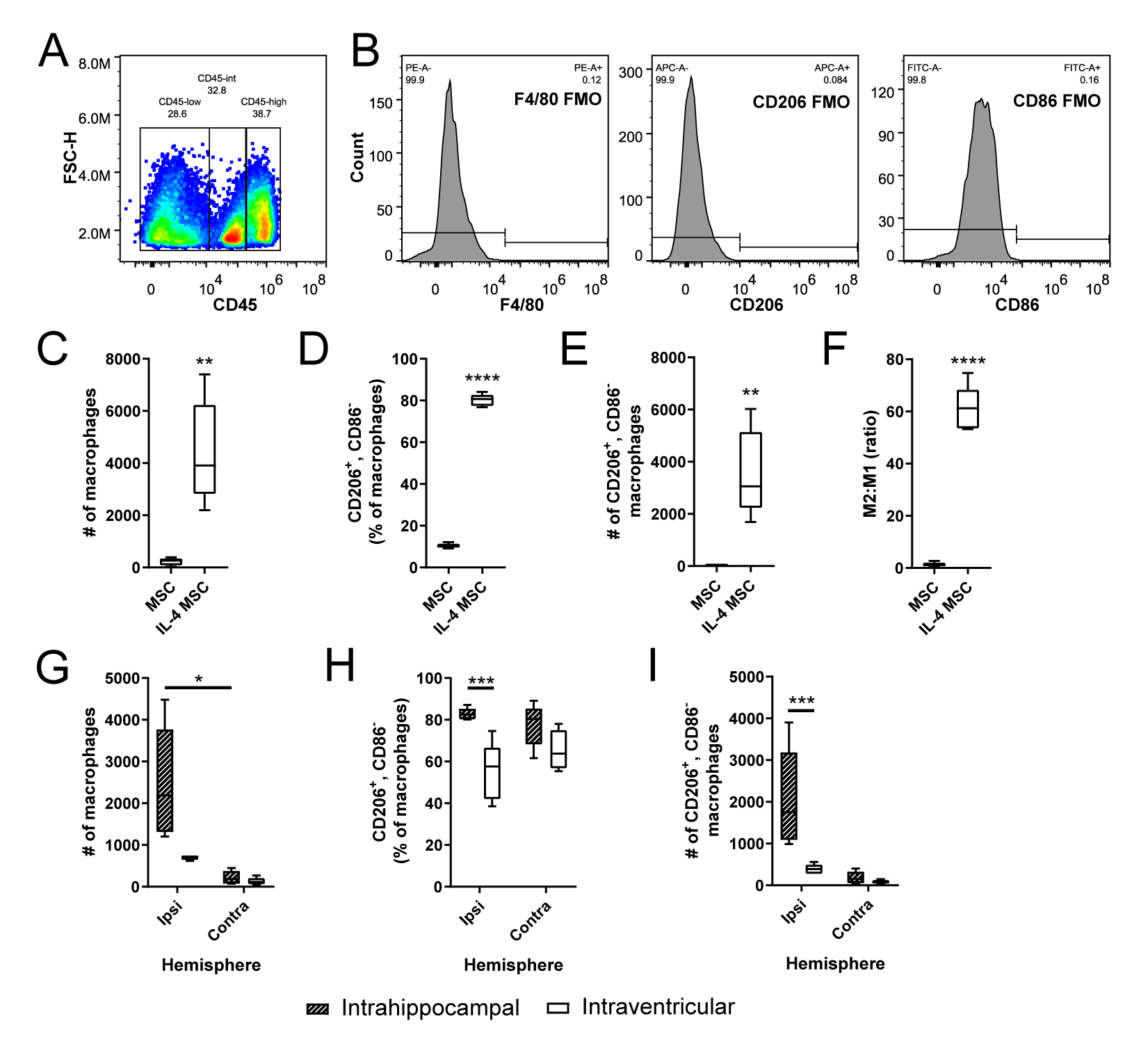

Supplement: Supplementary file 3 — Additional file 3: Figure S3. Macrophage polarization after closed head injury and treatment alterations through flow cytometry. (A) Pseudocolor flow plots demonstrating gating based on CD45 inensity to identify blood-derived leukocytes in the brain, CD45high. (B) Flow histograms of FMO controls to assist in gating. (C) – (F) Macrophage analysis 1 week after injury, 2 days after treatment with IL-4 MSCs producing greater IL-4; n = 5, N = 10. (C) Number of total macrophages in the ipsilateral hemisphere for each treatment group. (D) Percentage of all macrophages that possess M2-like phenotype. (E) Number of macrophages with M2-like phenotype. (F) Ratio of M2 to M2-like macrophages. (G) – (I) Macrophage analysis 1 week after injury, 2 days after treatment delivered into the left hippocampus or left lateral ventricle; n = 5, N = 10. (G) Total number of macrophages in the ipsilateral and contralateral hemispheres after either delivery modality. (H) Percent of total macrophages that possess an M2-like phenotype. (I) Number of M2-like macrophages in either hemisphere. Mean ± SD, Student’s t-tests or two-way ANOVAs with post-hoc Tukey’s; *p < 0.05, **p < 0.01, ***p < 0.001, ****p < 0.0001. [file 12974_2020_1860_MOESM3_ESM.tif]

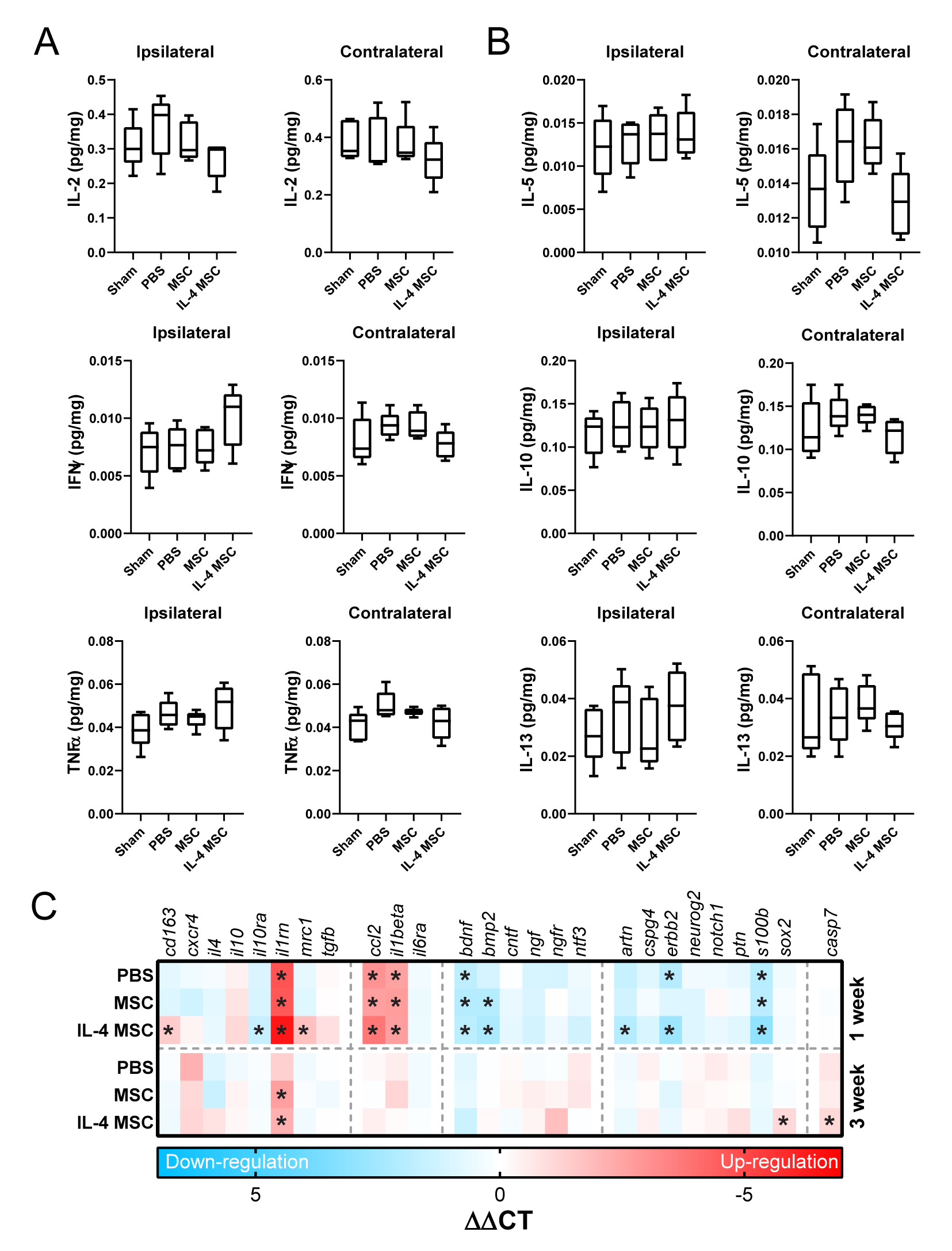

Supplement: Supplementary file 4 — Additional file 4: Figure S4. (A) – (B) Cytokine analysis of ex vivo injured and treated brain tissue at 1 week after injury. Sham mice or injured mice with day 5 treatment of either PBS, MSCs, or IL-4 MSCs; n = 5, N = 20. (A) Amount of inflammatory cytokine normalized to total protein per hemisphere (Interleukin 2, IL-2; Interferon-γ, IFNγ; and Tumor Necrosis Factor α, TNFα). (B) Amount of anti-inflammatory cytokine normalized to total protein per hemisphere (Interleukin 10, IL-10; Interleukin-13, IL-13; Interleukin-5, IL-5). Graphs display mean ± SD; one-way ANOVA carried out for each cytokine and hemisphere, with Bonferroni-Sidak correction for repeated testing. (C) Gene analysis at 1 week or 3 weeks after injury and day 5 treatment with either PBS, MSCs, or IL-4 MSCs (n = 5) and sham mice as biological controls (n = 10); N = 40. Heatmap of 26 genes demonstrating up- (red) or down- (blue) regulation of genes based on ΔΔCT values. Boxes with an asterisk (*) had a corrected p-value < 0.05 as reported by the analysis tool. [file 12974_2020_1860_MOESM4_ESM.tif]

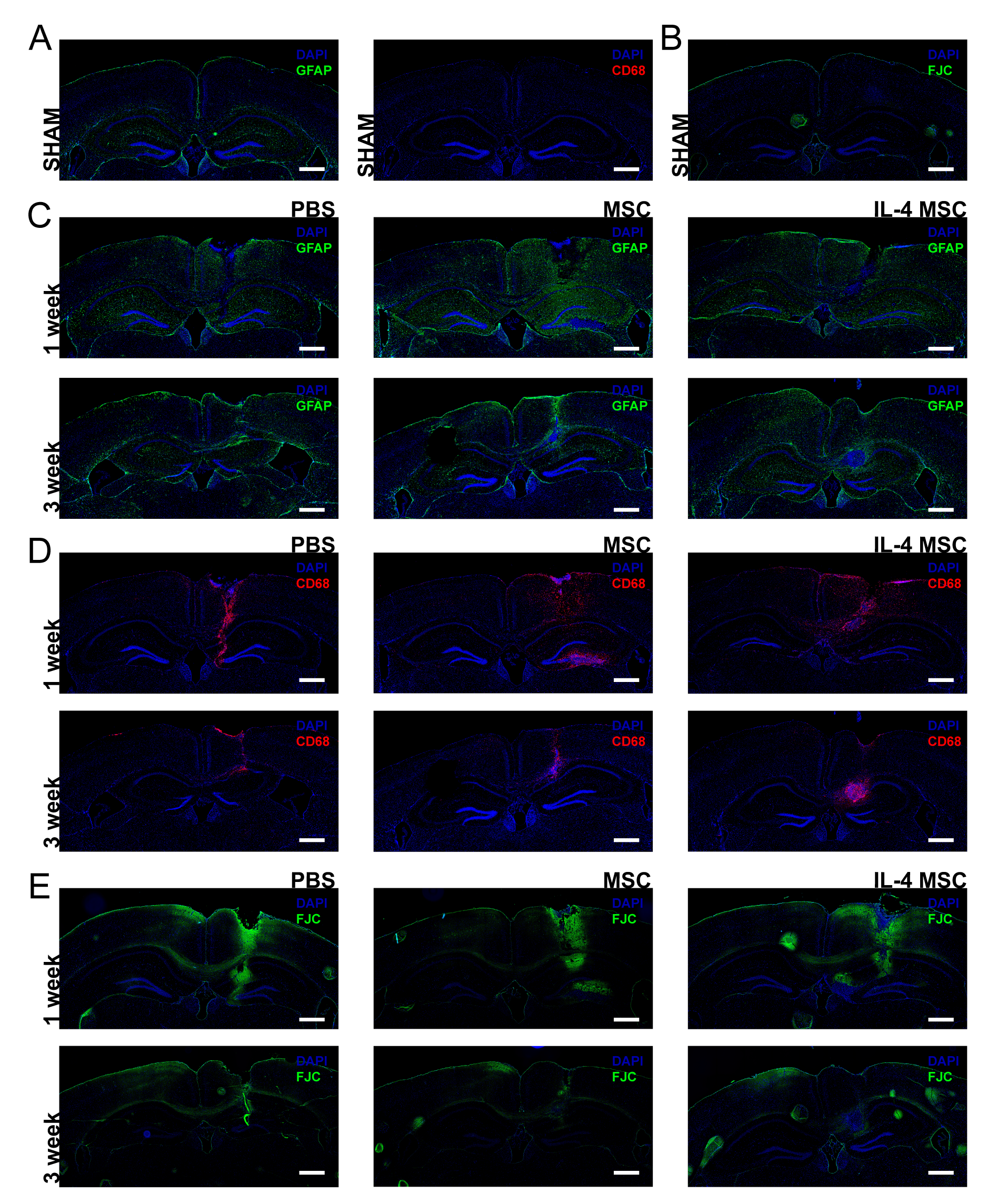

Supplement: Supplementary file 5 — Additional file 5: Figure S5. Representative immunohistochemical images of injured and treated brains at 1 and 3 weeks after injury. (A) Section from a sham group brain stained for DAPI, GFAP, and CD68. (B) Section from a sham group stained for DAPI and Fluoro-Jade C. (C)-(E) Representative sections from each injury/treatment group (columns) and timepoint (rows). (C) Stained for DAPI and GFAP. (D) Stained for DAPI and CD68. (E) Stained for DAPI and Fluoro-Jade C. [file 12974_2020_1860_MOESM5_ESM.tif]
